# Cellular CARD11 Inhibits the Fusogenic Activity of Newcastle Disease Virus via CBM Signalosome-Mediated Furin Reduction in Chicken Fibroblasts

**DOI:** 10.3389/fmicb.2021.607451

**Published:** 2021-02-02

**Authors:** Wenbin Wang, Qiaolin Wei, Qiqi Hao, Yajie Zhang, Yongshan Li, Youkun Bi, Zhongyuan Jin, Haijin Liu, Xuelan Liu, Zengqi Yang, Sa Xiao

**Affiliations:** ^1^College of Veterinary Medicine, Northwest A&F University, Yangling, China; ^2^Poultry Institute, Shandong Academy of Agricultural Science, Jinan, China

**Keywords:** Newcastle disease virus, CARD11, fusogenic activity, furin, CBM signalosome

## Abstract

Newcastle disease virus (NDV) causes an infectious disease that poses a major threat to poultry health. Our previous study identified a chicken brain-specific caspase recruitment domain-containing protein 11 (CARD11) that was upregulated in chicken neurons and inhibited NDV replication. This raises the question of whether CARD11 plays a role in inhibiting viruses in non-neural cells. Here, chicken fibroblasts were used as a non-neural cell model to investigate the role. CARD11 expression was not significantly upregulated by either velogenic or lentogenic NDV infection in chicken fibroblasts. Viral replication was decreased in DF-1 cells stably overexpressing CARD11, while viral growth was significantly increased in the CARD11-knockdown DF-1 cell line. Moreover, CARD11 colocalized with the viral *P* protein and aggregated around the fibroblast nucleus, suggesting that an interaction existed between CARD11 and the viral *P* protein; this interaction was further examined by suppressing viral RNA polymerase activity by using a minigenome assay. Viral replication was inhibited by CARD11 in fibroblasts, and this result was consistent with our previous report in chicken neurons. Importantly, CARD11 was observed to reduce the syncytia induced by either velogenic virus infection or viral haemagglutinin-neuraminidase (HN) and F cotransfection in fibroblasts. We found that CARD11 inhibited the expression of the host protease furin, which is essential for cleavage of the viral *F* protein to trigger fusogenic activity. Furthermore, the CARD11-Bcl10-MALT1 (CBM) signalosome was found to suppress furin expression, which resulted in a reduction in the cleavage efficiency of the viral *F* protein to further inhibit viral syncytia. Taken together, our findings mainly demonstrated a novel CARD11 inhibitory mechanism for viral fusogenic activity in chicken fibroblasts, and this mechanism explains the antiviral roles of this molecule in NDV pathogenesis.

## Introduction

Newcastle disease is caused by Newcastle disease virus (NDV) and is one of the most fatal diseases in poultry worldwide. NDV, known as avian paramyxovirus serotype 1 (APMV-1) or avian avulavirus 1, is currently classified into the genus *Orthoavulavirus* of subfamily *Avulavirinae* in family *Paramyxoviridae* of order *Mononegavirales*) (Rima et al., 2019). It comprises an enveloped, non-segmented, single-stranded, negative-sense RNA, which contains six genes encoding the nucleocapsid (NP), matrix protein (M), phosphoprotein (P), fusion protein (F), haemagglutinin-neuraminidase protein (HN), and large polymerase protein (L) (Wang et al., 2019). NP-RNA association with P-L forms an active ribonucleoprotein (RNP) complex that is necessary for transcription and replication (Zhang et al., 2017). NDV is generally classified into three pathotypes based on its clinical pathogenicity in chickens: lentogenic (avirulent or low virulent), mesogenic (moderately virulent), and velogenic (highly virulent) (Alexander, 2000).

Host cell proteins play essential roles in NDV pathogenesis. In our previous study, an NDV-induced brain-specific upregulated protein, caspase recruitment domain 11 (CARD11), was identified to inhibit viral replication through the CARD11 CC1 domain, and the viral *L* protein was found to competitively interact with the *X* domain of *P*, which hampers the *P*–*L* interaction and suppresses viral polymerase activity and viral replication (Wang et al., 2019). CARD11 comprehensively exists in human and animal tissues (Fagerberg et al., 2014; Yue et al., 2014). However, the role of CARD11 in NDV infection in non-neural cells is unknown. CARD11, also called CARMA1, is a multidomain scaffold protein that controls antigen-induced lymphocyte activation during the immune response (Bertin et al., 2001; Ramadas et al., 2011; Steinhardt et al., 2014). It interacts directly with B-cell lymphoma/leukemia 10 (Bcl10), and mucosa-associated lymphoid tissue lymphoma translocation protein 1 (MALT1) interacts with CARD11 only indirectly through Bcl10 to form the CARD11-Bcl10-MALT1 (CBM) complex (Wegener et al., 2006; Wegener and Krappmann, 2007; Roche et al., 2013; Juilland and Thome, 2016; Lork et al., 2019). Within the CBM signalosome, MALT1 plays a crucial role in translating the signal from the T-cell receptor (TCR) or B-cell receptor (BCR) to trigger activation of nuclear factor κ (NF-κB), c-Jun N-terminal kinase (JNK), and mammalian target of rapamycin (mTOR) (Gross et al., 2008; Lork et al., 2019). The mutation and dysregulation of CARD11 in lymphocytes causes diseases, such as various lymphomas and primary immunodeficiency (Moreno-Garcia et al., 2010; Scudiero et al., 2014; Turvey et al., 2014; Brohl et al., 2015).

The entry and propagation of virus into cells can induce a variety of cytopathic effects (CPEs). The typical CPE caused by membrane fusion of velogenic and mesogenic NDV or protease-treated lentogenic strains in susceptible cells is syncytium formation (Fan et al., 2017; Wang et al., 2017). Then the syncytia died and were exfoliated to produce plaques on cell monolayers. To achieve the fusogenic activity of NDV, *F*, and HN are two transmembrane glycoproteins responsible for viral envelope fusion with host cell membranes, allowing viral entry into the cytoplasm of target cells to cause viral spread and cell-cell membrane fusion (Gravel et al., 2011; Wang et al., 2017; Azarm and Lee, 2020). Both precursor *F*_0_ and cleaved F (F_1_ + F_2_) are present on NDV virions (Chambers and Samson, 1980; Yoshida et al., 1989). The F_0_ on virions may be further cleaved by cellular proteases to form two disulfide-linked subunits (F_1_ + F_2_) during virus infecting cells. Once the viral attachment protein HN binds to sialic acid-containing cell surface receptors, the conformation of cleaved F (F_1_ + F_2_) is refolded to catalyze membrane fusion, juxtaposing the target cell and viral membrane (Jardetzky and Lamb, 2014). The multibasic amino acid cleavage motif, “^112^(R/K)-R-Q-(R/K)-R↓F^117^” (R, arginine; K, lysine; Q, glutamine; G, glycine; F, phenylalanine; arrow, cleavage position; number, and residue position) in the precursor F_0_ is recognized by host proteases (Kawahara et al., 1992). For most paramyxoviruses, cellular proteases such as furin are responsible for cleaving the F_0_ protein in host cells. The furin, a ubiquitous cellular protease in a wide range of cells and tissues, prefers to recognize site “R-X-(R/K)-R↓” (X, any residue) that results in systemic infection of virulent virus (Murakami et al., 2001). Furin expression could be regulated by cellular signaling in HeLa cells (Kumar et al., 2010; Trebing et al., 2014).

In this study, we used the chicken fibroblast cell line DF-1 and chicken primary embryo fibroblasts (CEFs) as non-neural cell models to investigate the role of CARD11 in viral replication and syncytia. The expression of CARD11 was not significantly induced by velogenic and lentogenic strains in the infected CEFs and DF-1 cells. However, CARD11 was able to inhibit viral replication and suppress viral polymerase activity in fibroblasts, which was consistent with the results of our previous study (Wang et al., 2019). Importantly, we found that CARD11 inhibited the fusogenic activity of NDV by activating the CBM signalosome to suppress furin expression, resulting in reduced cleavage efficiency of the *F* protein. Our results provide novel insight into the mechanism of inhibiting fusogenic activity by CARD11 in non-neural cells.

## Materials and Methods

### Cells and Viruses

HEK293T cells (ATCC and CRL-11268), BHK-21 cells (ATCC and CCL-10), and DF-1 cells (ATCC and CRL-12203) were cultured in Dulbecco’s modified Eagle’s medium (DMEM) (Life Technologies, United States) containing 10% fetal bovine serum (FBS) (SeraPro, Germany). CEFs were prepared from 10-day-old specific pathogen-free (SPF) embryonated chicken embryos. Briefly, the trunks were washed with sterile PBS, cut into pieces, digested with 0.25% trypsin without EDTA (Life Technologies, United States) at room temperature for 15 min and stirred at low speed using a magnetic stirring apparatus. The cells were centrifuged at 1500 rpm for 5 min and suspended in sterile PBS. The cells were centrifuged at 1500 rpm for 5 min again, and DMEM was supplemented with 10% FBS and 1% penicillin/streptomycin (P/S). The cell resuspension solution was filtered through 149, 74, and 47 μm cell strainers and seeded in a cell culture dish. All cells were maintained at 37°C in a 5% CO_2_ atmosphere. The F48E9 and LaSota strains of NDV were obtained from the China Institute of Veterinary Drug Control. The velogenic strain F48E9 is used for standard vaccine challenge in China. The lentogenic strain LaSota is globally used for poultry vaccines. These viruses were propagated in 9- to 11-day-old SPF embryonated chicken eggs purchased from Jinan SAIS Poultry Co., Ltd. (China). All viruses were stored at −80°C.

### Real-Time qPCR

Total RNA of cell samples was isolated using TRI Gene Reagent (GenStar, China). To detect the relative mRNA expression of chicken CARD11 and furin, 5 μg of isolated RNA was reverse-transcribed using the StarScript II First-strand cDNA Synthesis Kit (GenStar, China) with oligo (dT) primers. Real-time qPCR was performed on an Applied Biosystem QuantStudi^TM^ 6 Flex Real-Time PCR System (Applied Biosystems, United States) using EvaGreen 2 × qPCR MasterMix-ROX (Applied Biological Materials, United States). Primers are shown in Table 1. The relative mRNA levels of genes were normalized to that of 28S rRNA and were calculated by the comparative threshold cycle (ΔΔCt) method. The ratios of gene expression between the treatment and control samples were calculated as 2^–Δ^ ^Δ^ ^*Ct*^.

**TABLE 1:** Primers in this study.

Gene name	Forward primer (5′-3′)	Reverse primer (5′-3′)
Lenti-CARD11	*TCTAGA*ATGAGCACCCAAGGAGGAGAGC	*GCGGCCGC*TTAGAGCTGATCTTCATCTATCCAGATGG
qPCR CARD11	GCTTCTGACACGGCAGCATCT	CATTTCCTTTAGCCCAATCCTC
qPCR chicken furin	CAGCTGCGTTCTCATTGTCG	AGGCAGTCCTCTCATTGTGC
28S rRNA	GGTATGGGCCCGACGCT	CCGATGCCGACGCTCAT

*^RT^represents reverse transcription. Restriction enzyme sites are in italic.*

### Lentivirus production

To knockdown CARD11, the chicken U6 promoter (chU6, GenBank no. DQ531569) was synthesized (Synbio Tech, China), digested with *Spe*I and *Bgl*II and cloned into the lentiviral vector pCD513B-1, which contains a GFP gene. This construct was then digested with *Spe*I and *Bam*HI to generate pCD513B-1-chU6. Lentiviral vectors encoding CARD11-specific shRNAs (shRNA1 and shRNA2) and a non-silencing negative control (NC) siRNA were cloned into the *Bam*HI and *Eco*RI sites. The NC sequence was 5′-GATCCCGTGATCTTCACCGACAAGATTTCAAGAGAATCT TGTCGGTGAAGATCACGTTTTTTG-3′, and the shRNA1 and shRNA2 sequences were 5′-GATCCGCAGATGACT CCTCCACATCATTCAAGAGATGATGTGGAGGAGTCATCT GCTTTTTTG-3′ and 5′-GATCCGCTCCTTCAGTGCACTCTT CTTTCAAGAGAAGAAGAGTGCACTGAAGGAGCTTTTTT G-3′, respectively. The lentiviral pseudoparticles were produced as described previously (Chen et al., 2016). Subjection of all viruses to freeze-thaw cycles was avoided before use.

### Establishment of stable DF-1 cell lines

For the construction of the CARD11-overexpressing plasmid, CARD11 was amplified from DF-1 cells using a pair of primers (Table 1) and ligated into pTSB-CMV-MCS-SBP-3Flag-tRFP-F2A-Puro (Hanyang Biological Technology, China) via the *Xba*I and *Not*I sites. To establish DF-1 cells overexpressing CARD11, DF-1 cells seeded in 6-well plates were transfected with pTSB-CMV-MCS-SBP-3Flag-tRFP-F2A-Puro and pTSB-CMV-CARD11-SBP-3Flag-tRFP-F2A-Puro. After transfection for 8 h, the medium was replaced with fresh culture medium and cultured for 48 h. Then, the cells were selected with 9 μg/ml puromycin (Sigma-Aldrich, United States). The selected cell lines were named “control” and “ovCARD11”. For CARD11-knockdown DF-1 cell lines (namely, NC, shRNA1, and shRNA2), DF-1 cells were seeded in 6-well plates at a density of 5 × 10^5^ cells per well and transduced with pseudoparticles using 8 μg/ml polybrene (Sigma-Aldrich, United States). After 12 h of incubation, the medium was replaced with fresh culture medium. The cells were selected with 9 μg/ml puromycin and harvested after an additional 48 h. All monoclonal cells were purified after being selected with puromycin three times.

### Viral Infection

DF-1 cells or established cell lines were infected with the F48E9 or LaSota strain at multiplicities of infection (MOIs) of 0.01 and 1, respectively, at 37°C for 1 h; then, cells were washed three times with sterile PBS and cultured in DMEM containing 2% FBS. The culture medium was harvested at different hours postinfection (hpi). The CPE was visualized under an Olympus IX73 inverted research microscope (Japan). The viral titers of F48E9-infected cells were determined by a 50% tissue culture infective dose (TCID_50_/mL) assay, while those of LaSota-infected DF-1 cells were determined by an immunofluorescence assay (IFA) in DF-1 cells (Wang et al., 2019). The titer of TCID_50_/mL was calculated according to the method established by Reed and Muench (Guo et al., 2018).

### Minigenome Assay

The minigenome (MG) system of the LaSota strain was constructed as previously described (Wang et al., 2019). A 5-plasmid system of MG was used: pCAGGS-T7, pMG-Fluc, pcDNA3-HA-NP, pcDNA3-HA-P, and pCAGGS-L at a ratio (μg) of 5:5:2:2:1 that were cotransfected with pTSB-CMV-CARD11-SBP-3Flag-tRFP-F2A-Puro (2 μg) or pTSB-CMV-MCS-SBP-3Flag-tRFP-F2A-Puro (2 μg) into DF-1 cells. The relative luciferase activity was normalized to that of Renilla luciferase by transfecting pRL-SV40-N (Beyotime Biotechnology, China). The cells were harvested at 36 h posttransfection, and the relative luciferase activity was measured by the Spark^®^ multimode microplate reader (TECAN, Switzerland) and calculated using the ratio of relative light units according to the manufacturer’s manual (Beyotime Biotechnology, China).

### IFA

Newcastle disease virus-infected CEFs and DF-1 cells were fixed with 4% paraformaldehyde in PBS for 15 min, washed three times with PBS, and permeabilized with 0.1% Triton X-100 for 10 min at room temperature. After blocking with PBS containing 1% bovine serum albumin (BSA) for 1 h, the cells were incubated with the primary antibodies anti-CARD11 mouse pAb (1:100) and anti-P (LaSota) guinea pig pAb (1:200) (Wang et al., 2019) in PBS at 4°C overnight. After washing with PBS three times, the samples were incubated with the secondary antibodies goat anti-guinea pig IgG/FITC (Bioss, China) and goat anti-mouse IgG H&L (Alexa Fluor 647, preadsorbed, Abcam, and ab150115) for 1 h at room temperature. The cell nuclei were stained with 4′,6′-diamidino-2-phenylindole (DAPI, Beyotime Biotechnology, China). All cells were observed and photographed under a confocal microscope (Olympus FV3000, Japan).

### Trypsin Recovery Aassay

The trypsin concentration is optimized in the Control and ovCARD11 cells. The Control and ovCARD11 cells are seeded in 12-well plates at the same cell number and then infected with F48E9 (0.01 MOI) or cotransfected with pCAGGS-HN (1 μg) and pCAGGS-GST-F (1 μg). The analysis is set into two groups. Group 1: The cells were observed at 24 hpi or 24 h post transfection without trypsin treatment. Group 2: The cells were treated with trypsin for 12 h, and were observed at 24 hpi or 24 h post transfection. The numbers of nuclei in 40 fusion areas were counted to determine the average syncytia size after 24 h of infection and coinfection as described below.

### Chicken NF-κB (chNF-κB) Reporter Assay

The chicken NF-κB reporter assay was performed as previously described (Wang et al., 2019). Briefly, the established DF-1 cell lines (control, ovCARD11, NC, and shRNA1) were cotransfected with pchNF-κB-TA-luc and pRL-SV40-N at a ratio of 10:1. At 36 h posttransfection, the cells were harvested to measure the firefly luciferase and Renilla luciferase activity using the Spark^®^ multimode microplate reader (TECAN, Switzerland), and the relative luciferase activity normalized to the Renilla luciferase activity was calculated according to the manufacturer’s manual (Beyotime Biotechnology).

### Treatment of Inhibitors

The NF-κB inhibitors BAY 11-7082 for IκBα, BMS-345541 for IKKα and IKKβ, and MI-2 for MALT1 (MedChemExpress, United States) were dissolved in DMSO to provide 10 mM stock solutions and stored at −20°C. MI-2 is an irreversible MALT1 inhibitor. DF-1 cells were observed and tested for cell survival after incubation with increasing concentrations (1, 5, 8, 10, and 20 μM of BAY 11-7082; 0.1, 1, 5, and 10 μM of BMS-345541; and 1 and 2 μM of MI-2 or DMSO) for 24 h. Control and ovCARD11 cells were seeded in 12-well plates and treated with the optimal concentration of inhibitors for 24 h. Then, the cells were infected with F48E9 (0.01 MOI) or transfected with pCAGGS-HN (1 μg) and pCAGGS-GST-F (1 μg). The culture supernatants were harvested at 24 and 36 hpi for titration of NDV. The numbers of nuclei in 40 fusion areas were counted to determine the average syncytia size after 36 h of infection as described below.

### Flow Cytometry

The cell toxicity for optimizing the concentration of inhibitors in DF-1 cells was tested by FITC-Annexin V and PI Apoptosis Kit (US Everbright, Inc., China). The cells treated with the inhibitors for 24 h were washed twice with sterile PBS and digested by 0.25% trypsin without EDTA (Gibco, United States) for 2 min until all cells were separated. Then, the cells were washed twice in PBS and centrifuged at 300 g for 5 min at 4°C. Finally, the cells were resuspended in 100 μl of 1 × binding buffer containing 5 μl FITC-Annexin V and 2 μl PI and incubated at room temperature for 15 min. The prepared samples were analyzed using a FACS Calibur instrument (BD FACS Aria TM III, United States).

### Western Blot Assay

The CARD11 expression in CEFs or DF-1 cells was detected by immunoprecipitation enrichment (IPE) with an anti-CARD11 mouse pAb as previously described (Wang et al., 2019). The inhibitor-treated BHK-21 cells and other cell samples were lysed with 1 × SDS-PAGE loading buffer and then boiled for 10 min. After centrifugation, the supernatants were collected and subjected to 12% SDS-PAGE. The proteins were transferred onto a polyvinylidene fluoride membrane (PVDF) (Millipore, United States). Immunoblotting was performed using the following primary antibodies: anti-DYKDDDDK-Tag (3B9) mouse mAb (Abcam, United States), anti-furin rabbit pAb (Proteintech, United States), anti-GST mouse mAb (Sungene Biotech, China), anti-NP (LaSota) guinea pig pAb (Guo et al., 2018), anti-P (LaSota) guinea pig pAb (Wang et al., 2019) and anti-HN (LaSota) mouse mAb (prepared by us) or anti-β-tubulin (3G7) mouse mAb (Sungene Biotech, China). The secondary antibodies included goat anti-mouse IgG conjugated to horseradish peroxidase (HRP) (Sungene Biotech, China) and goat anti-rabbit IgG antibody (H&L)-HRP (ZETA, China). An enhanced chemiluminescent (ECL) peroxidase substrate (Millipore) was used for the detection of proteins by using the Tanon 5,200 Chemiluminescent Imaging System (Tanon, China).

### Plaque Assay

BHK-21 cells in 24-well plates (∼4 × 10^4^ cells per well) were transfected with pTSB-CMV-MCS-SBP-3Flag-tRFP-F2A-Puro (1 μg) or pTSB-CMV-CARD11-SBP-3Flag-tRFP-F2A-Puro (1 μg) and then infected with the same amount of F48E9. DF-1 cells (∼4 × 10^4^ cells per well) were treated with three inhibitors for 24 h and then infected with F48E9. The supernatants were replaced with DMEM containing 2% FBS and 1% methyl cellulose (Solarbio, China) after 1 h incubation, and the cells were incubated for an additional 3–4 days. Next, the overlay medium was removed and washed three times in PBS and then fixed with methanol for 25 min. The cells were washed three times in PBS and stained with crystal violet (Solarbio, China). The diameter of each plaque was measured using ImageJ software, and the average size was calculated.

### Fusogenic Activity Assay

NDV-infected DF-1 cells and CARD11-overexpressing or CARD11-knockdown DF-1 cell lines cotransfected with HN and F in 6-well plates and 12-well plates were measured for fusogenic activity. The numbers of nuclei in 40 fusion areas were counted to determine the average syncytia size after 24 or 36 h. The average syncytia size was calculated as the ratio of the total number of nuclei in multinucleated cells to the total number of nuclei in the field.

### Statistical Analysis

Two-tailed Student’s *t*-test was used to estimate the statistical significance between two columns by GraphPad Prism 5. All data from three independent experiments are presented as the mean ± standard deviation (SD) of triplicate samples (*n* = 3); *p* < 0.05 was considered statistically significant. ns, not significant, ^∗^*p* < 0.05; ^∗∗^*p* < 0.01; ^∗∗∗^*p* < 0.001.

## Results

### CARD11 Expression Is Not Significantly Affected in NDV-Infected Chicken Fibroblasts

**FIGURE 1:**
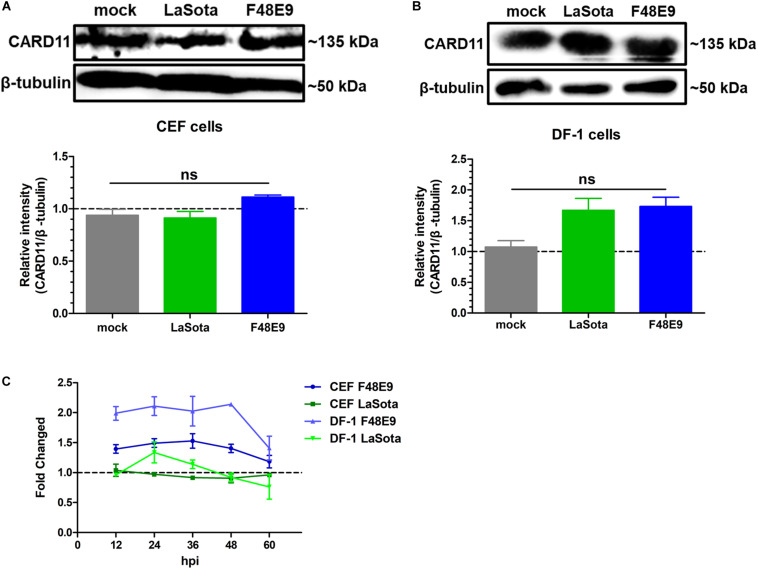
CARD11 was not significantly upregulated by NDV infection in chicken fibroblasts. **(A,B)** CEFs **(A)** and DF-1 **(B)** cells seeded in 100-mm plates were infected with F48E9 (0.01 MOI) or LaSota (1 MOI), harvested at 36 hpi and detected using the IPE method. The expression levels of CARD11 relative to that of β-tubulin were analyzed by densitometry via ImageJ software. **(C)** The relative mRNA expression of CARD11 in viral-treated CEFs and DF-1 cells at different hpi was determined by qPCR normalized to the mRNA expression of 28S rRNA. All representative data from three independent experiments (mean ± SD) were analyzed with a two-tailed Student’s *t*-test. ns, not significant.

In our previous report, CARD11 expression was brain-specifically upregulated by NDV infection in chicken neurons (Wang et al., 2019). Here, we investigated whether the expression level of CARD11 was affected by NDV infection in chicken fibroblasts. We found that CARD11 expression was not significantly upregulated in either virulent strain F48E9- or lentogenic strain LaSota-infected CEFs (Figure 1A) or DF-1 cells (Figure 1B). The relative CARD11 mRNA level in the NDV-infected CEFs and DF-1 cells at 0 hpi had no difference with the mock-infected cells. Based on this, the relative CARD11 mRNA level in the F48E9-infected CEFs from 12 to 48 hpi was compared with that in the mock-infected cells, and it was increased by only 0.5-fold and that in the infected DF-1 cells was increased by only one-fold (Figure 1C). These results were consistent with the previous transcriptomic profiles of NDV-infected CEFs (Munir et al., 2005), where CARD11 was not differentially upregulated by NDV.

### CARD11 Overexpression Inhibits Viral Replication

**FIGURE 2:**
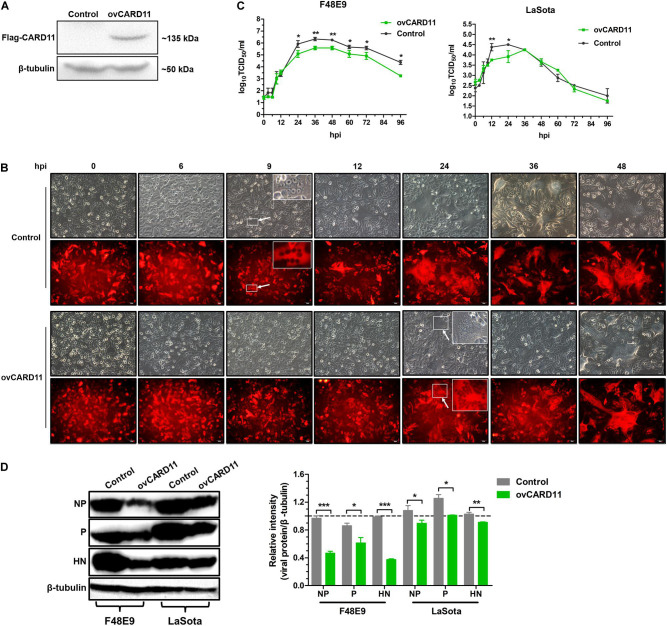
CARD11 overexpression inhibits viral replication. **(A)** The protein expression of CARD11 was detected with an anti-Flag mouse mAb through western blot in selected control and ovCARD11 cells after three rounds of puromycin selection. **(B)** CPE in F48E9 (0.01 MOI)-infected control and ovCARD11 cells. The syncytia indicated by white arrows were zoomed on the top right corner. Scale bar = 50 μm. **(C)** The viral titers in F48E9 (0.01 MOI) and LaSota (1 MOI)-infected control and ovCARD11 cells. **(D)** The viral NP, P, and HN proteins in F48E9 (0.01 MOI) and LaSota (1 MOI)-infected control and ovCARD11 cells at 36 hpi were detected by a western blot assay. The expression levels relative to that of β-tubulin were analyzed by densitometry via ImageJ software. The results are presented as the mean ± SD of three independent experiments and were analyzed by two-tailed Student’s *t*-test. ns, not significant, *p < 0.05, **p < 0.01, ***p < 0.001.

Next, we verified whether CARD11 played an inhibitory role in NDV replication in DF-1 cells. A DF-1 cell line stably overexpressing CARD11 (ovCARD11) and a NC cell line (control) were established by transfection with a CARD11 expression vector (Figure 2A). The CPE and syncytia appeared at 24 hpi in the F48E9-infected ovCARD11 cells that delayed for nearly 15 h compared to the infected control cells. Large syncytia appeared at 48 hpi in the F48E9-infected ovCARD11 cells (Figure 2B). The growth titers of F48E9 in the ovCARD11 cells were significantly decreased compared with those in the control cells at 24 hpi (Figure 2C). All F48E9-infected cells were dead at 72 hpi. The syncytia infected with velogenic strain F48E9 were severe at 36 hpi and cells began to die. No matter the control group or the experimental group, viral particles released in the supernatant were decreased with cells dying, and reduced at 37°C from 60 hpi and 96 hpi. LaSota is unable to cause syncytia. The viral titers of LaSota in the ovCARD11 cells were only dramatically decreased at 12 and 24 hpi compared with the Control cells (Figure 2C). Simultaneously, the expression of the viral proteins, especially the NP and HN proteins, in the infected cells was suppressed in NDV-infected ovCARD11 cells (Figure 2D). These results showed that the overexpression of CARD11 delayed the occurrence of CPE and decreased viral replication in fibroblasts.

### CARD11 Depletion Increases Viral Replication

**FIGURE 3:**
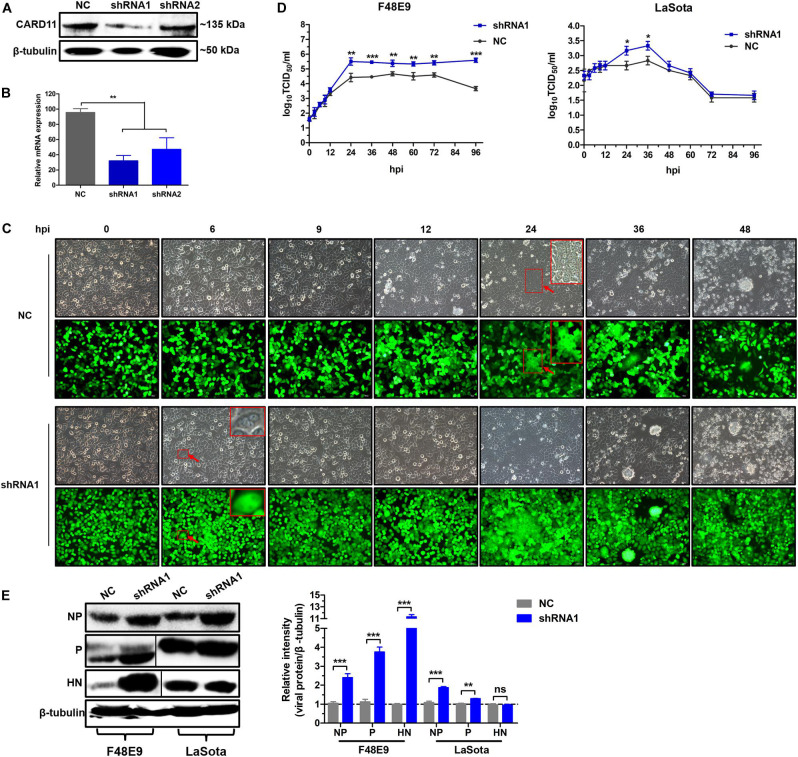
CARD11 depletion increases viral replication. **(A,B)** The expression of CARD11 in knockdown DF-1 cell lines. The data were normalized to those of 28S rRNA. **(C)** CPEs in the F48E9-infected CARD11-knockdown DF-1 cell line. The syncytia indicated by red arrows were zoomed on the top right corner. Scale bar = 50 μm. **(D)** Growth titer of F48E9 (0.01 MOI) and LaSota (1 MOI) in CARD11/shRNA1 cells. **(E)** The viral NP, P, and HN proteins in F48E9 (0.01 MOI) and LaSota (1 MOI)-infected control and ovCARD11 cells at 36 hpi were detected by western blotting. The expression levels relative to that of β-tubulin were analyzed by densitometry via ImageJ software. Data are presented as the mean ± SD of three independent experiments and were analyzed by two-tailed Student’s *t-*test. ns, not significant, *p < 0.05, **p < 0.01, ***p < 0.001.

To test whether the depletion of CARD11 affected the replication of NDV, the CARD11-knockdown cell lines shRNA1 and shRNA2 were established by lentivirus-shRNA transduction. The interference efficiency of shRNA1 was more effective than that of shRNA2 in the expression levels of either protein (Figure 3A) or mRNA (Figure 3B). F48E9 infection caused CPE and syncytia in shRNA1 cells as early as 6 hpi. However, the occurrence of CPE and syncytia in the infected NC cells was delayed for nearly 18 h (Figure 3C), indicating that CARD11 depletion enhanced viral CPE during early infection. All infected shRNA1 cells were dead at 72 hpi, but the infected NC cells were dead at 96 hpi, which led to the dramatically increased viral titer in shRNA1 cells. The titers of F48E9 were significantly higher in the shRNA1 cells than in the NC cells after 24 hpi (Figure 3D). The titers of LaSota were increased in the shRNA1 cells at only 24 and 36 hpi (Figure 3D). The growth kinetics of F48E9 were much higher than those of LaSota in the shRNA1 cells. Consistently, the levels of the F48E9 and LaSota viral proteins, especially the NP and *P* proteins, in the shRNA1 cells were significantly increased compared to those in the NC cells (Figure 3E), implying that the inhibitory role of CARD11 was more effective against the virulent virus than against the avirulent virus. These results suggested that CARD11 knockdown increased the occurrence of CPE and viral replication.

### CARD11 Colocalizes With Viral *P* and Inhibits Viral Replication

**FIGURE 4:**
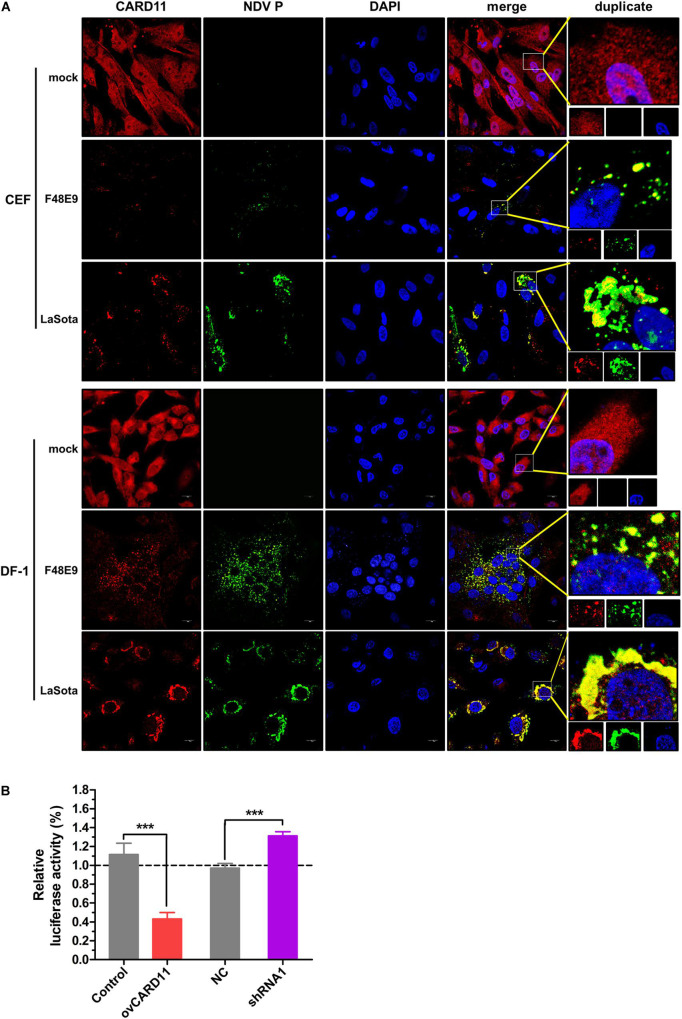
Subcellular colocalization of CARD11 and the viral *P* protein in chicken fibroblasts. **(A)** CARD11 colocalized with the viral *P* protein in F48E9 (MOI = 0.01) and LaSota (MOI = 1)-infected CEFs and DF-1 cells. CARD11 (red), *P* protein (green), and the nucleus (blue) were observed by confocal microscopy. Scale bar = 10 μm. **(B)** Inhibition of viral RNA polymerase activity by CARD11 overexpression using a three-plasmid system. The DF-1 cell lines were cotransfected with pCAGGS-T7, pCMV-NP-P-L, and MG-Fluc at a ratio of 2:1:1. All the cells above were cotransfected with pRL-SV40-N expressing Renilla luciferase as a normalizing standard and lysed at 36 h posttransfection. Representative data, shown as the mean ± SD (*n* = 3), were analyzed by two-tailed Student’s *t*-test. ***p < 0.001.

CARD11 was able to colocalize with viral P in NDV-infected chicken primary neuronal cells as previously described (Wang et al., 2019). Here, we attempted to examine whether CARD11 colocalized with viral *P* in chicken fibroblasts. Endogenous CARD11 was observed throughout the cytoplasm distribution in mock-infected CEFs and DF-1 cells (Figure 4A) and subcellularly colocalized with the viral *P* protein that aggregated around the nucleus in both F48E9- and LaSota-infected cells (Figure 4A), suggesting that CARD11 interacts with the viral *P* protein in fibroblasts, which is consistent with our previous results in neurons (Wang et al., 2019). Moreover, we examined whether this interaction of CARD11 and viral *P* could suppress the activity of viral RNA polymerase in shRNA1 cells by using a MG system assay (Figure 4B). The results revealed that CARD11 could inhibit viral RNA polymerase activity in fibroblasts.

### CARD11 Reduces NDV-Induced Syncytium Formation Without Altering the Fusogenic Property of Progeny Viruses

**FIGURE 5:**
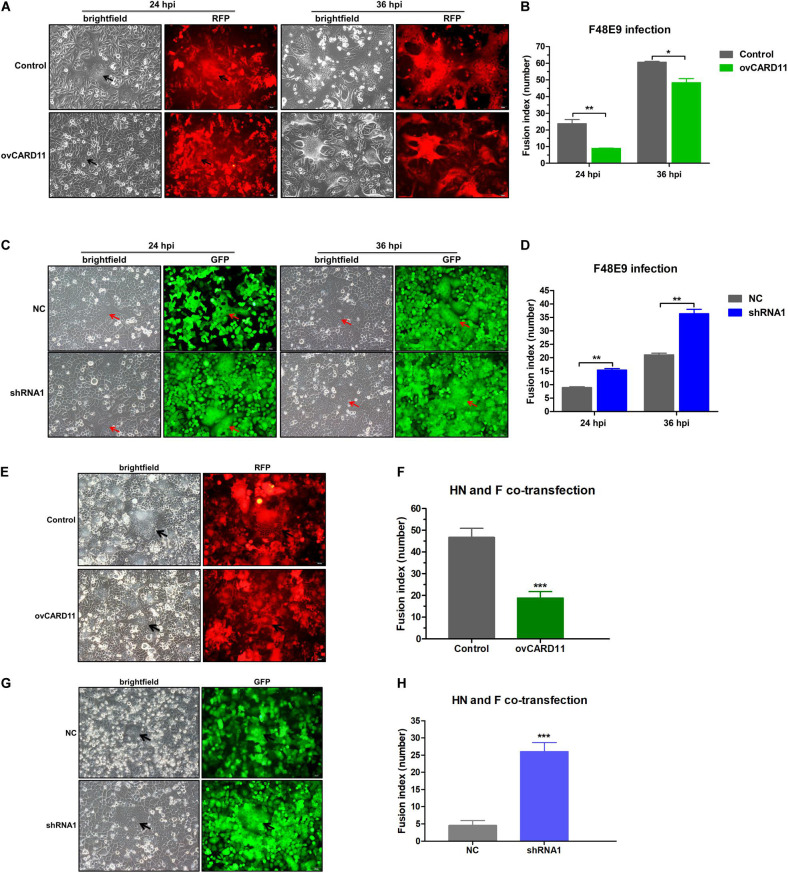
CARD11 inhibits viral fusogenic activity. **(A,C)** Syncytia formation in the F48E9 (0.01 MOI)-infected CARD11-overexpressing and CARD11-knockdown cell lines were observed at 24 and 36 hpi. The black and red arrows indicate the syncytia. Scale bar = 50 μm. **(B,D)** The numbers of nuclei in 40 fusion areas were counted to determine the average syncytia size at 24 and 36 hpi. **(E,G)** CARD11-overexpressing or CARD11-knockdown DF-1 cells in 6-well plates were cotransfected with pCAGGS-HN (1 μg) and pCAGGS-GST-F (1 μg). The syncytia were observed after 24 h. The arrows indicate the syncytia. Scale bar = 50 μm. **(F,H)** The numbers of nuclei in 40 fusion areas were counted to determine the average syncytia size after 24 h. All the average syncytia sizes were calculated as the ratio of the total number of nuclei in multinuclear cells to the total number of nuclei in the field. Data are presented as the mean ± SD of three independent experiments and were analyzed by two-tailed Student’s *t*-test. *p < 0.05, **p < 0.01, ***p < 0.001.

**FIGURE 6:**
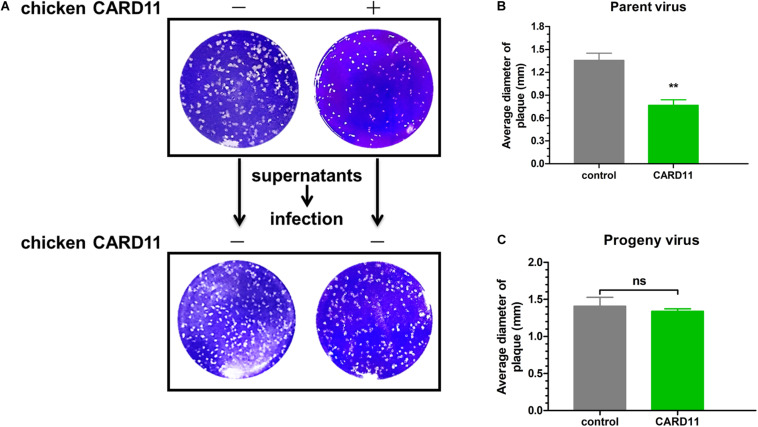
CARD11 does not alter the fusogenic properties of progeny viruses. **(A)** Plaque formation induced by parent and progeny viruses in BHK-21 cells. The cells transfected with pTSB-CMV-CARD11-SBP-3Flag-tRFP-F2A-Puro or empty vector were infected with F48E9 (0.01 MOI). The supernatant was harvested at 36 hpi and then infected with BHK-21 cells for the plaque assay. **(B,C)** The average plaque area was analyzed using ImageJ software, as shown on the right. All the data are presented as the mean ± SD of three independent experiments and were analyzed by two-tailed Student’s *t*-test. ns, not significant, **p < 0.01.

Virulent NDV can cause syncytium formation in cells. Using velogenic F48E9, we found that syncytia were inhibited in infected ovCARD11 cells at 24 and 36 hpi (Figures 5A,B). Conversely, syncytia were enhanced in F48E9-infected shRNA1 cells (Figures 5C,D). Moreover, syncytia were also significantly inhibited in ovCARD11 cells cotransfected with viral *F* and HN (Figures 5E,F), whereas syncytia were enhanced in shRNA1 cells cotransfected with viral *F* and HN (Figures 5G,H). Similarly, the plaque sizes in infected chicken BHK-21 cells transiently overexpressing CARD11 were smaller than those in the control cells (Figures 6A,B). Furthermore, we investigated whether the progeny viruses yielded from the cells transiently overexpressing CARD11 changed their ability to form plaques and grow. The plaque sizes of progeny viruses produced from the control and CARD11-overexpressing BHK-21 cells showed no difference in the plaque assay (Figures 6A,C). These results indicated that CARD11 could reduce the syncytium formation of velogenic virus but did not alter the ability of the progeny viruses to produce plaques in the cells.

### CARD11 Inhibits the Fusogenic Activity of NDV by Suppressing Furin Expression

**FIGURE 7:**
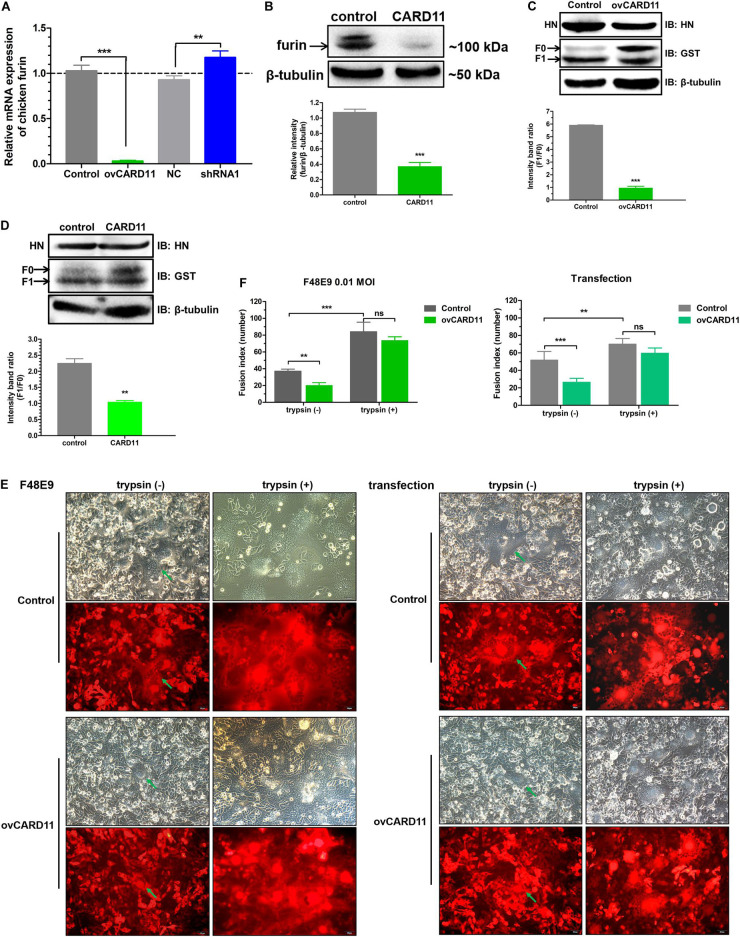
CARD11 suppresses furin expression to inhibit the cleavage efficiency of the viral *F* protein. **(A)** The relative mRNA expression of furin in DF-1 cell lines. **(B)** The expression of furin protease in chicken BHK-21 cells transiently overexpressing CARD11. The expression levels relative to that of β-tubulin were analyzed by densitometry with ImageJ software. **(C,D)** The expression of HN and F in ovCARD11 cells **(C)** and chicken CARD11-overexpressing BHK-21 cells **(D)**. BHK-21 cells in 12-well plates were first transfected with pTSB-CMV-CARD11-SBP-3Flag-tRFP-F2A-Puro and empty vector, and 24 h later, they were cotransfected with pCAGGS-HN (1 μg) and pCAGGS-GST-F (1 μg). The viral proteins were detected with an anti-GST mouse mAb and an anti-LaSota HN guinea pig pAb. β-tubulin was used as a protein loading control. The ratio of *F*_1_/*F*_0_ was analyzed by densitometry with ImageJ software. Syncytia formation **(E)** and, the average syncytia size **(F)** in the F48E9 (0.01 MOI)-infected and pCAGGS-HN (1 μg) and pCAGGS-GST-F (1 μg) cotransfected CARD11-overexpressing cell lines were observed and determined at 24 hpi with or without trypsin treatment. The green arrows indicate the syncytia. Scale bar = 50 μm. Data are presented as the mean ± SD of three independent experiments and were analyzed by two-tailed Student’s *t-*test. ns, not significant, **p < 0.01, ***p < 0.001.

Furin is a ubiquitous intracellular protease in most cell types and can proteolytically cleave the *F* protein of virulent NDV, resulting in syncytium formation (Kim et al., 2013). We examined whether CARD11 regulates the expression of furin and affects the fusogenic activity of NDV in DF-1 cells. The mRNA expression of furin was dramatically decreased in the ovCARD11 cells and increased in the shRNA1 cells (Figure 7A). To examine the expression level of cellular furin, BHK-21 cells were used instead of DF-1 cells for detection by an anti-mouse furin antibody, which was chosen because an anti-chicken furin antibody was not available. Furin was obviously inhibited in chicken CARD11-overexpressing BHK-21 cells (Figure 7B). Glycosylation and other post-translational modifications can make the pre-pro furin run at 100-110 kDa, the mature furin at 98-95 kDa and shed furin at 90 kDa. Moreover, the cleavage efficiency of viral *F* protein (*F*_1_/*F*_0_) was reduced in the ovCARD11 cell line (Figure 7C) and chicken CARD11-overexpressing BHK-21 cells (Figure 7D). Furthermore, the trypsin recovery assay showed that the average syncytia size was reduced in infected ovCARD11 cells at 24 h compared with Control cells without trypsin treatment no matter F48E9 infection and co-transfection. However, the average syncytia size of cells cultured with trypsin (7 μg/mL) for 12 h became larger, and there was no significance between Control and ovCARD11 cells (Figures 7E,F). In addition, the trypsin accelerated the syncytia formation (Figure 7F). This indicated that the fusion level could be restored by supplementation of trypsin although CARD11 reduced the level of cellular furin. These results suggested that CARD11 inhibited the fusogenic activity by suppressing furin expression to reduce the cleavage efficiency of the viral *F* protein.

### CBM Signalosome Activation Inhibits the Viral Fusogenic Activity

**FIGURE 8:**
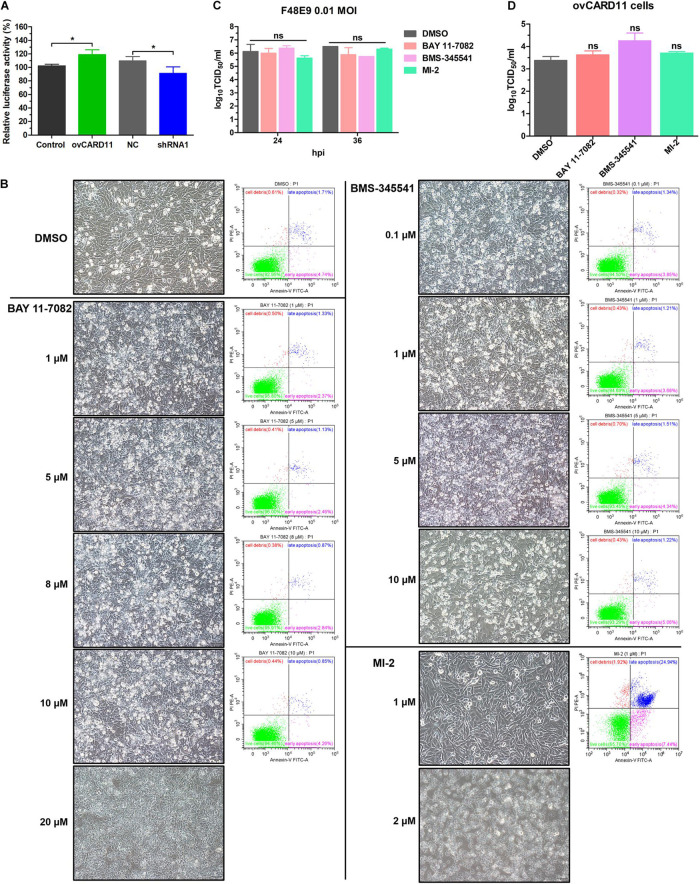
Select the optimal inhibitor concentrations. **(A)** NF-κB activation in ovCARD11 cells or shRNA1 cells. **(B)** Optimal concentration of inhibitors in DF-1 cells. DF-1 cells in 12-well plates were treated with BAY 11-7082, BMS-345541, and MI-2 at different concentrations for 24 h. These cells were observed and harvested to detect cell survival using flow cytometry. Scale bar = 50 μm. **(C,D)** The viral titer of F48E9 (MOI = 0.01) in inhibitor-treated DF-1 **(C)** and ovCARD11 cells **(D)**. The supernatant at 24 and 36 hpi was harvested for TCID_50_/mL. Data are presented as the mean ± SD of three independent experiments and were analyzed by two-tailed Student’s *t*-test. ns, not significant, *p < 0.05.

**FIGURE 9:**
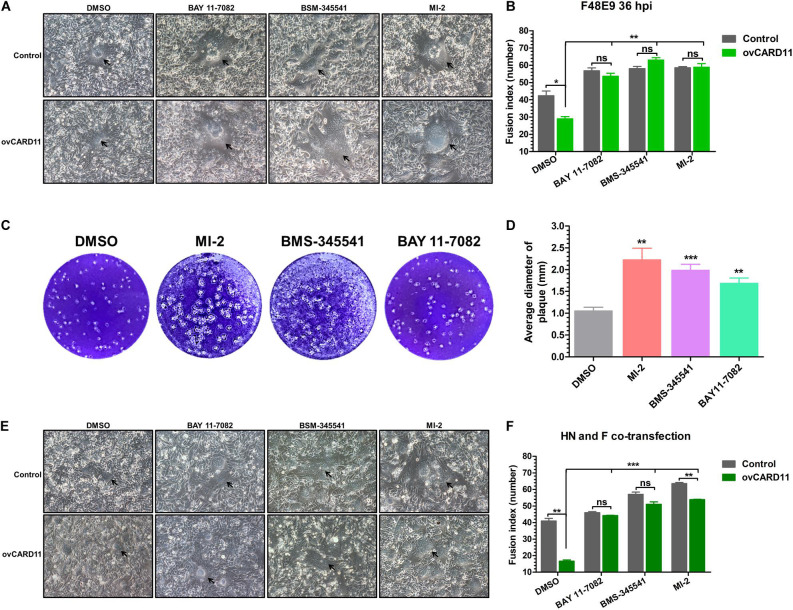
Activation of the CBM signalosome inhibits fusogenic activity. **(A)** The syncytial formation of F48E9 cells at 36 hpi in inhibitor-treated control and ovCARD11 cells. The black arrows indicate the syncytia. Scale bar = 50 μm. **(B)** The numbers of nuclei in 40 fusion areas were counted to determine the average syncytia size. **(C)** Plaque formation in F48E9-infected inhibitor-treated DF-1 cells. **(D)** The average plaque area was analyzed using ImageJ software. **(E)** The syncytial formation of HN and F cotransfection at 36 hpi in the inhibitor-treated control and ovCARD11 cells. The black arrows indicate the syncytia. Scale bar = 50 μm. **(F)** The numbers of nuclei in 40 fusion areas were counted to determine the average syncytia size. All the data are presented as the mean ± SD of three independent experiments and were analyzed by two-tailed Student’s *t-*test. ns, not significant, *p < 0.05, **p < 0.01, ***p < 0.001.

Caspase recruitment domain 11 serves as a nucleation center for the CBM signalosome following TCR or BCR stimulation. The proteinase MALT1 of the CBM complex is believed to be an essential step for triggering the activation of the following pathways: canonical NF-κB, mTOK, and JNK (Lu et al., 2018). We hypothesized that CARD11 might act through CBM signaling to affect viral fusogenic activity. First, we used a luciferase reporter system with a chNF-κB promoter to assess NF-κB activation. The luciferase activity was increased in the transfected ovCARD11 cells. Conversely, the luciferase activity was decreased in transfected shRNA1 cells (Figure 8A). The data revealed that CARD11 could induce NF-κB activation in fibroblasts. Next, to investigate the CBM signaling involved in the fusogenic activity of NDV, we used three inhibitors, MI-2 for MALT1, BAY 11-7082 for NF-κB, and BMS-345541 for IKK, in the cells. To optimize the concentrations of the three inhibitors in DF-1 cells, their cytotoxicities were determined by CPE observation and an apoptosis assay in the treated DF-1 cells. BAY 11-7082 and BMS-345541 at 10 μM and MI-2 at 1 μM were chosen for the following experiments because their maximum effective concentrations caused minimal cytotoxicity (Figure 8B). BAY 11-7082 and BMS-345541 were able to inhibit the activity of luciferase in a chNF-κB reporter assay in DF-1 cells examined in previous research (Wang et al., 2019). With the inhibitor treatments, the viral titers showed no difference in NDV-infected DF-1 cells (Figure 8C) or ovCARD11 cells (Figure 8D), and these results were consistent with our previous data in neuronal cells. To test whether the three inhibitors affect viral fusogenic activity, syncytium formation was analyzed by their fusion indexes and a plaque assay. The syncytia of the F48E9 infection in the inhibitor-treated control and ovCARD11 cells were larger than those in the DMSO-treated cells without inhibitors (Figures 9A,B). The average viral plaque diameter in three assays with inhibitor-treated DF-1 cells, especially those with MI-2, was much larger than that in three assays with control cells (Figures 9C,D). The syncytia of HN and F cotransfection in the inhibitor-treated control and ovCARD11 cells were also larger than those in the DMSO-treated cells without inhibitors (Figures 9E,F). These results clearly indicated that the CBM signalosome is involved in inhibiting viral fusogenic activity.

### CBM Signalosome Suppresses Furin Expression

**FIGURE 10:**
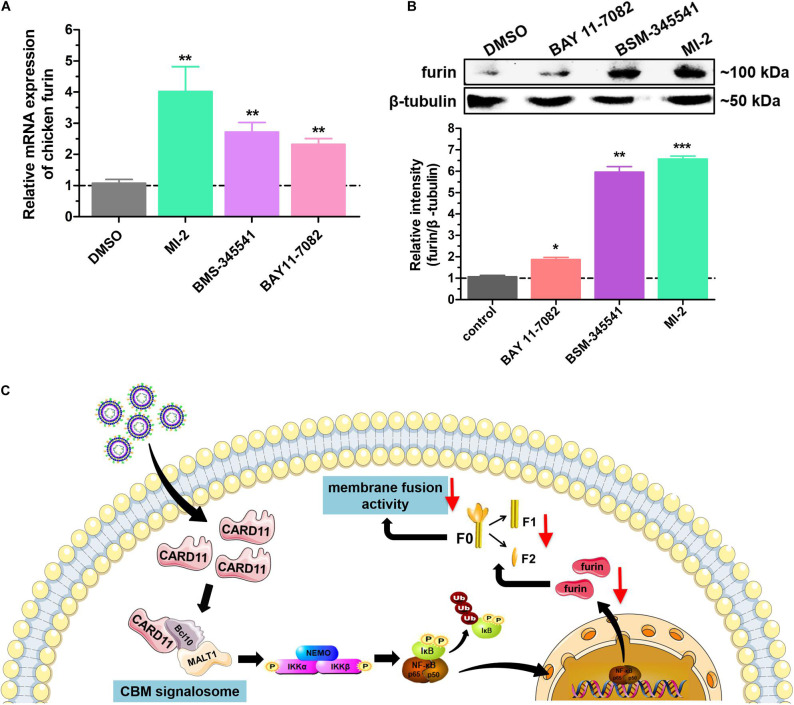
Activation of the CBM signalosome suppresses furin expression. **(A)** The relative mRNA expression of furin in inhibitor-treated DF-1 cells. **(B)** The expression of furin protease in inhibitor-treated BHK-21 cells. The expression levels relative to that of β-tubulin were analyzed by densitometry. **(C)** The schematic diagram of the CARD11 inhibitory mechanism for viral fusogenic activity in chicken fibroblasts. Data are presented as the mean ± SD of three independent experiments and were analyzed by two-tailed Student’s *t* test. *p < 0.05, **p < 0.01, ***p < 0.001.

We next raised the question of whether the CBM signalosome affects expression of furin. To answer this question, we analyzed furin expression under treatment with three inhibitors by RT-qPCR and western blot analyses. The mRNA expression of furin was significantly increased in inhibitor-treated DF-1 cells (Figure 10A). The protein expression of furin was enhanced by treatment with the inhibitor MI-2 in BHK-21 cells (Figure 10B). The results suggested that CBM signalosome activation could suppress furin expression, resulting in fusogenic activity inhibition in the cells.

## Discussion

CARD11 is brain-specifically upregulated by NDV infection in chickens, which reduces viral replication and the CPEs in infected chicken neuronal cells as previously described (Wang et al., 2019). To determine the roles of CARD11 in chicken non-neural cells, we used chicken CEF and DF-1 fibroblasts as cell models. Our findings showed that CARD11 expression was not significantly upregulated in NDV-infected CEFs or DF-1 cells. However, CARD11 still inhibited viral replication in the overexpressed DF-1 cell lines. As in chicken neuronal cells, the CARD11-induced inhibition of viral replication in the fibroblasts was likely mediated by the interaction of CARD11 and the viral *P* protein to suppress viral RNA polymerase activity. Remarkably, syncytium formation can be obviously caused by velogenic NDV in fibroblasts, whereas it cannot be observed in neuronal cells due to the dispersed cell growth of neurons. We further found that viral syncytia formation or fusogenic activity was inhibited by CARD11 through CBM signalosome activation to suppress host protease furin expression, resulting in a reduction in the cleavage efficiency of the viral *F* protein.

Phosphorylation upon antigen receptor engagement is crucial for maximal CARD11 activity (Brenner et al., 2009; Bedsaul et al., 2018). In the resting cells, CARD11 intramolecular interactions between the linker region and CARD-CC domain to keep CARD11 in an auto-inhibited conformation preventing its CARD domain to interact with BCL10 CARD domains (Lork et al., 2019). Once TCR/BCR is activated by antigen, PKCβ/θ phosphorylated CARD11 in its linker region on S552 and S645, causing a conformational change from an inactive “locked” conformation to an active “open” conformation, which is crucial to recruit BCL10 and then activate NF-κB pathway (Matsumoto et al., 2005; Sommer et al., 2005; Eitelhuber et al., 2011; Thys et al., 2018). Other kinases, IKKβ and hematopoietic progenitor kinase 1 (HPK1) were able to phosphorylate CARD11 on S555 and S551, respectively, leading to an optimal NF-κB activation (Shinohara et al., 2007; Brenner et al., 2009). In this study, unlike in chicken neuronal cells, CARD11 was not significantly upregulated in NDV-infected chicken fibroblasts, but it could still inhibit NDV replication and viral fusogenic activity. The possibility is that NDV may facilitate the phosphorylation of CARD11 resulting in the assembly of CBM complex and NF-κB activation. This hypothesis needs to be further verified for detecting CARD11 phosphorylation level with its phosphorylation-specific antibody.

The CBM signalosome is primarily involved in signal transduction downstream of receptors (Hu and Sun, 2016; Lu et al., 2018). MALT1, as a protease, plays a bridging role that leads to the activation of NF-κB, JNK, and mTOR in lymphocytes (Lu et al., 2018). Canonical NF-κB activation is mediated by the activation of the IκB kinase (IKK) complex, which consists of two catalytic subunits, IKKα and IKKβ, and a regulatory subunit NF-κB essential modulator (NEMO, also known as IKKγ) (Zhang et al., 2017). MALT1 acts as a scaffold to facilitate the oligomerization and activation of TRAF6 or TGFβ-activated kinase 1 (TAK1) to phosphorylate IKKβ (Noels et al., 2007; Oeckinghaus et al., 2007; Meininger and Krappmann, 2016). This allows NF-κB to translocate into the nucleus to initiate target gene transcription. Meanwhile, TAK1 recruits MKK7, and selective JNK2 phosphorylation leads to the accumulation and phosphorylation of c-Jun, which regulates lymphocyte proliferation as part of the AP-1 transcription factor complex (Wan et al., 2006; Blonska et al., 2007). Recent studies have demonstrated that MALT1 has the ability to associate with mTOR, and its paracaspase activity mediates the phosphorylation of ribosomal protein S6, a target of mTORC1, ultimately impacting metabolic programming (Hamilton et al., 2014). In this study, the three inhibitors MI-2, BMS-345541 and BAY 11-7082 affected the different inhibitory roles in furin expression and the fusogenic activity of NDV (Figures 9, 10). This finding was associated with previous studies showing that NF-κB activation could inhibit the expression of the cellular protease furin (Kumar et al., 2010; Trebing et al., 2014). It is worth noting that the two NF-κB inhibitors, BMS-345541 and BAY 11-7082, have different effects on furin because of the different target molecules and mechanisms. BAY 11-7082 decreases NF-κB by inhibiting TNFα-induced phosphorylation of IκBα, and BMS-345541 is a selective inhibitor of the catalytic subunits of IKK (IKKα and IKKβ) by binding at an allosteric site of IKK. Above all, once CBM or MALT1 was blocked, downstream signaling molecules, such as NF-κB, were completely blocked, implying that the CBM signalosome played a critical role in regulating the fusogenic activity of NDV.

Caspase recruitment domain 11 is a globally expressed protein in animals (Fagerberg et al., 2014; Yue et al., 2014). The basic expression of CARD11 in chicken tissues had no significant differences (data not shown). Previously, CARD11 was identified as being neuron-specifically upregulated in NDV-infected chicken brains and primary neurons (Wang et al., 2019). In this study, although CARD11 expression was not significantly upregulated in NDV-infected chicken fibroblasts, it still had the ability to inhibit NDV. Obviously, CARD11 might exhibit a similar antiviral mechanism in neuronal and fibroblast cell types, but its inhibitory effect might be different in various tissue cells.

In conclusion, our findings demonstrated that CARD11 inhibited NDV replication and viral syncytium formation in chicken CEFs and DF-1 cells. The interaction between CARD11 and the viral *P* protein suppressing viral RNA polymerase activity might be involved in the inhibitory mechanism of viral replication in fibroblasts. The inhibition of viral fusogenic activity could occur through the activation of the CBM signalosome to reduce furin expression, mediating the decreasing cleavage efficiency of the viral *F* protein. Our study revealed a novel inhibitory mechanism of CARD11 in the viral fusogenic activity of NDV in fibroblasts. This would improve the understanding of the antiviral roles of CARD11 in NDV infection.

## Data Availability Statement

The original contributions presented in the study are included in the article/Supplementary Material, further inquiries can be directed to the corresponding author/s.

## Author Contributions

WW was responsible for experiment design, data analysis, and wrote the manuscript. QW, QH, YZ, and YL performed the experiments. YB, ZJ, HL, and XL contributed to reagents and materials. ZY was responsible for suggestion during the experiments performance. SX was responsible for revising the manuscript. All authors read and approved the final manuscript.

## Conflict of Interest

The authors declare that the research was conducted in the absence of any commercial or financial relationships that could be construed as a potential conflict of interest.
