# Supplementary material for: Cellular CARD11 Inhibits the Fusogenic Activity of Newcastle Disease Virus via CBM Signalosome-Mediated Furin Reduction in Chicken Fibroblasts
Source: Front Microbiol. 2021 Feb 2;12:607451. doi: 10.3389/fmicb.2021.607451 (PMC7884349; doi:10.3389/fmicb.2021.607451)
Supplement: Supplementary file 1 [file Data_Sheet_1.PDF]

Supplementary Material

Supplementary Figures

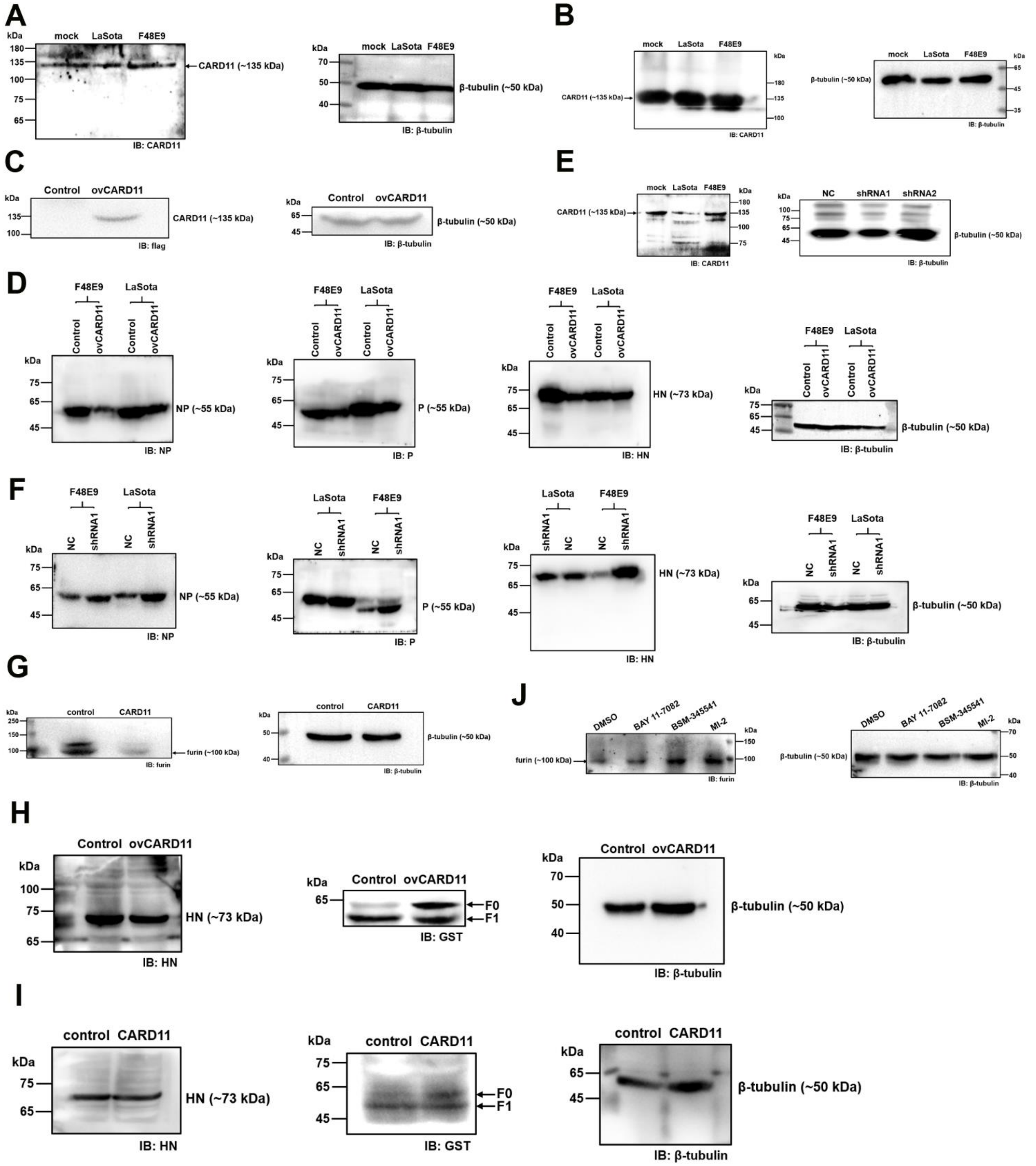

Supplementary Figure 1. The original images of western blot assay without any modification in the manuscript. S1A corresponds to Figure 1A, S1B to Figure 1B, S1C to Figure 2A, S1D to Figure 2D, S1E to Figure 3A, S1F to Figure 3E, S1G to Figure 7B, S1H to Figure 7C, S1I to Figure 7D, S1J to Figure 10B in the manuscript, respectively. These gels were cut appropriately according to the molecular weight of proteins and were transferred onto PVDF membrane, so that these blots were only showed within limits.
